# *Catha edulis* Leaves: Morphological Characterization and Anti-Inflammatory Properties in an In Vitro Model of Gastritis

**DOI:** 10.3390/plants13111538

**Published:** 2024-06-01

**Authors:** Andrea Magnavacca, Claudia Giuliani, Gabriella Roda, Stefano Piazza, Giulia Martinelli, Carola Pozzoli, Nicole Maranta, Alessio Papini, Martina Bottoni, Eleonora Casagni, Michele Dei Cas, Gelsomina Fico, Mario Dell’Agli, Enrico Sangiovanni

**Affiliations:** 1 Department of Pharmacological and Biomolecular Sciences “Rodolfo Paoletti”, University of Milan, Via Balzaretti 9, 20133 Milan, Italy; and93ream@gmail.com (A.M.); giulia.martinelli@unimi.it (G.M.); carola.pozzoli@unimi.it (C.P.); nicole.maranta@unimi.it (N.M.); mario.dellagli@unimi.it (M.D.); enrico.sangiovanni@unimi.it (E.S.); 2 Department of Pharmaceutical Sciences, University of Milan, Via Mangiagalli 25, 20133 Milan, Italy; claudia.giuliani@unimi.it (C.G.); gabriella.roda@unimi.it (G.R.); martina.bottoni@unimi.it (M.B.); eleonora.casagni@unimi.it (E.C.); gelsomina.fico@unimi.it (G.F.); 3 Ghirardi Botanical Garden, Department of Pharmaceutical Sciences, University of Milan, Via Religione 25, Toscolano Maderno, 25088 Brescia, Italy; 4 Department of Biology, University of Florence, Via La Pira 4, 50120 Florence, Italy; alessio.papini@unifi.it; 5 Department of Health Sciences, University of Milan, Via di Rudinì 8, 20146 Milan, Italy; michele.deicas@unimi.it

**Keywords:** khat, leaf microscopy, gastric disorders, GES-1, inflammation, cathine, cathinone

## Abstract

Khat leaves, indigenous to eastern Africa, have been chewed for centuries for their stimulant effects, attributed to alkaloids such as cathinone and cathine. Although associated with gastric disorders like gastritis and gastro-oesophageal reflux disease, the underlying molecular mechanisms remain unclear. This study aimed to examine the morpho-anatomy of khat leaves using light microscopy and histochemistry and to assess the effects of leaf extracts and alkaloids on human gastric epithelial cells (GES-1). The study identified specific cells in the palisade–spongy transition zone as storage sites for psychoactive alkaloids. Leaf extracts were prepared by mimicking the chewing process, including a prolonged salivary phase followed by a gastric phase. Cytotoxicity and cell viability were evaluated using LDH and MTT assays, respectively. Additionally, the impact on IL-8 secretion, a key chemokine in gastric inflammation, was analysed under normal and TNF-α-stimulated conditions. The results showed no increase in cytotoxicity up to 250 µg/mL. However, there was a significant decrease in cell metabolism and a reduction in both basal and TNF-α-induced IL-8 secretion, but cathinone and cathine were inactive. These findings suggest that khat may not directly cause the gastric issues reported in the literature, which would rather be attributed to other confounding factors, highlighting the need for further research to clarify its biological impacts.

## 1. Introduction

*Catha edulis* (Vahl) Endl. (Celastraceae), known as khat, is an evergreen shrub or small tree that can reach 2–3 m in height. It is native to the Horn of Africa and distributed in the Arabian peninsula, where chewing young, fresh leaves has a long history as a social custom, dating back thousands of years [1]. The stem is straight and slender, with thin branches presenting 10–16 leaves arranged in opposite pairs at the distal portion; the leaf insertion is occasionally alternate at the proximal one. The leaves, slightly coriaceous and briefly petiolate, are ovate–lanceolate with an entire margin, an acute and symmetrical base, and an acute apex; they are bright green and glossy with a prominent yellow or reddish-brown midrib. The flowers are small, pentamer and white, and arranged in axillary cymes. The fruits are three-valved capsules, and the seeds display an aril and a small wing at the base [2].

In the areas of origin, khat is traditionally offered to welcome and entertain guests [1] on the basis of the belief that chewing khat leaves reduces the feeling of hunger and provides relief from fatigue by increasing vigilance. During a session, an individual can chew up to one bundle (about 200 g) of fresh leaves, swallowing the juice of the vegetable material and spitting out the residues. The initial pharmacological effects include euphoria and increase in communication abilities and self-confidence, excitability, arousal, elevation of blood pressure, and augmented alertness, soon replaced by irritability, depression, anorexia, and insomnia [1,3,4,5]. The stimulant properties are due to the presence of psychoactive alkaloids (cathine and cathinone, Figure S1) that possess sympathomimetic activity similar to amphetamine compounds.

Currently, the plant is mainly cultivated in Ethiopia, Kenya, and Yemen [6,7], where it is also used in folk medicine. However, the evidence concerning the ethnomedicinal value of khat is scarce. Some of the documented ethnopharmacological human and veterinary indications of khat leaves and roots include diarrhoea, gonorrhoea, helminthiasis, toothache, erectile dysfunction, cough and influenza [8], asthma, pneumonia and airway diseases [9], heartburn and stomachache [2]. The primary method of administration is by chewing fresh vegetable material or preparing infusions of fresh or dried leaves. Nevertheless, evidence of limited antimicrobial efficacy (especially against Gram negative bacteria and yeasts) and toxicity at high doses might limit the rational ethnopharmacological use of khat leaves [10].

In the native areas, the increase in khat consumption is strictly related to the economic advantages that the harvesting of the plant has brought to small traders. The trade of khat is legal in different African countries where its use is culturally significant, e.g., Kenya, Somalia, Djibouti, Ethiopia, Uganda, and Yemen, whereas it is illegal in Eritrea, Kuwait, Saudi Arabia, Sudan, Tanzania, and Zambia [11]. Khat consumption poses a potential risk to human health; thus, increasing the knowledge regarding adverse effects related to its abuse is a relevant issue [12,13]. Khat chewing has been associated with psychological dependence [14], while sub-chronic oral administration in mice has shown enhanced locomotor activity, reduced social interaction, and impaired cognitive function, suggesting that long-term use of khat might be related to schizophrenic-like syndromes [15]. Khat consumption has also been associated with cancer of the digestive tract and gastrointestinal inflammatory disorders [16,17], in open contrast with the traditional use against stomachache. However, the molecular mechanisms underlying these alleged harmful effects have not been elucidated yet.

Moving on from these premises, the first aims of this study were: (i) to deepen the understanding of leaf morpho-anatomy by means of light microscopy in combination with a histochemical approach intended to describe the sites of storage of alkaloids; and (ii) to investigate the risk of gastric damage related to khat leaf consumption by evaluating its cytotoxicity on gastric epithelial cells in vitro. However, the traditional use of khat drove us to aim toward a parallel investigation of its anti-inflammatory effects. Khat leaves, both fresh and dried, were subjected to an in vitro extraction–digestion protocol which included a prolonged salivary phase, mimicking the chewing process, followed by a gastric phase. In parallel, a hydromethanolic extract of fresh leaves was prepared to investigate the polar fraction of the phytocomplex. The biological activities of the extracts were evaluated in vitro on a human gastric epithelial cell line (GES-1), assessing cell viability and the effect on IL-8 secretion, the main chemokine involved in gastric inflammation [18]. Finally, the biological effects of the isolated molecules cathinone and cathine were evaluated.

## 2. Results

### 2.1. Micromorphological Survey

This survey involved both young and fully expanded leaves. The overall leaf morpho-anatomical features proved homogeneous across all the examined samples.

**Figure 1:**
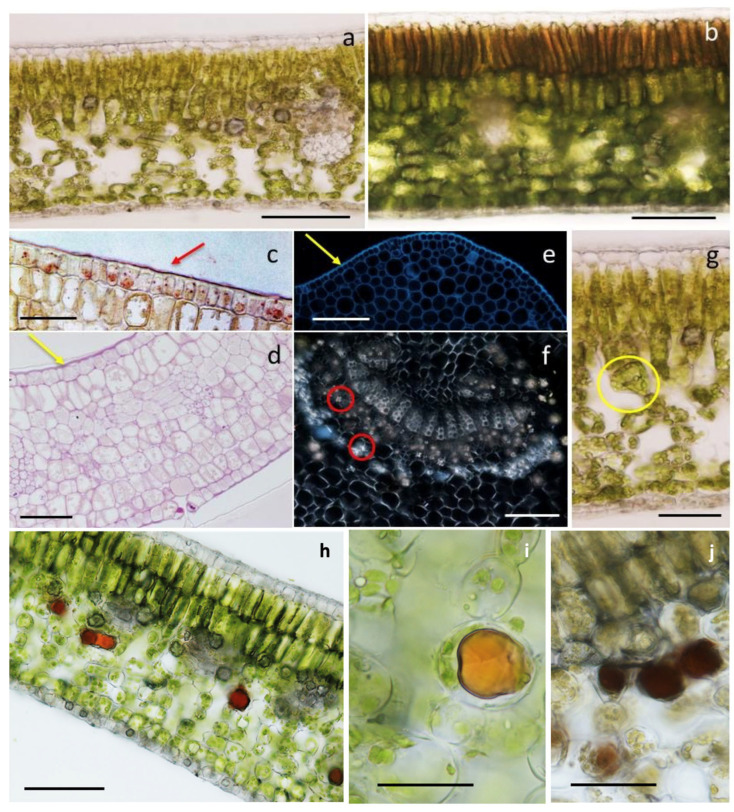
(**a,b**) Cross-section of a fully expanded leaf (**a**) and a young apical leaf (**b**). (**c**) Cuticle (red arrow) covering the epidermis, Sudan III/IV. (**d**) Cross-section of a young leaf, with thickened external tangential walls (yellow arrow), periodic acid Schiff stain. (**e**) Cross-section of a young leaf at the midrib; note the thickening of the outer tangential walls of the epidermal cells (yellow arrow), calcofluor. (**f**) Druses associated with the vascular system (red circles), polarized light. (**g**) Funnel-shaped cells (yellow circle) at the transitional region between the palisade and spongy mesophylls. (**h**–**j**) Results of the histochemical survey showing cross-sections of leaves treated with Wagner’s reagent for alkaloids (**h**,**i**) and ferric trichloride for polyphenols (**j**). Scale bars: (**a**,**b**,**f**–**h**) = 50 µm; (**c**–**e**,**i**) = 25 µm; (**j**)= 15 µm.

Figure 1 shows the cross-section of a leaf. The adaxial epidermis consisted of a single layer of polygonal cells covered by a thick cuticle (Figure 1a–c), and the external periclinal walls were thicker than the others for the massive deposition of cellulose and callose (Figure 1d,e). No stomata were observed.

The mesophyll was clearly differentiated in palisade and spongy parenchyma in all the observed samples of the fully expanded leaves (Figure 1a). Conversely, the young apical leaves displayed more homogeneous mesophyll patterns (Figure 1b), like a typical palisade organization: 4–5 layers of compact cells. The cells of the layer underlying the upper epidermis were polygonal in shape, while those of the other layers were globular–elliptic.

In the fully expanded leaf, the palisade consisted of 2–3 layers of globose cells with thin walls (Figure 1a). Sporadic calcium oxalate inclusions in the form of druses were occasionally observed in the innermost layer. At this level, the palisade organization was often discontinuous due to the presence of small intercellular spaces.

The spongy mesophyll consisted of 3–4 layers of globular or elongated cells with large and regularly arranged intercellular spaces. Sporadic druses, occupying almost the whole cell volume, were observed (Figure 1a); the druses were generally associated with the vascular system and were particularly abundant at the midrib (Figure 1f).

In the transitional region between the palisade and the spongy parenchymas, peculiar funnel-shaped cells were observed (Figure 1g).

The lower epidermis (Figure 1a,d) consisted of a single layer of polygonal cells and thickened external periclinal walls. The epidermis was covered by a thick cuticle with the presence of stomata and occasional calcium oxalate crystals.

Concerning the histochemical techniques, the application of Wagner’s and Dragendorff’s reagents, specific for alkaloids, showed intense positive results, highlighting the vacuolar content of some spongy mesophyll cells generally located at the transitional region with the palisade parenchyma (Figure 1h,i). The presence of the sites of storage of alkaloids was also observed in the leaf petiole, at the level of some cells of the cortical parenchyma. In the leaves, the treatment with the ferric trichloride test, specific for polyphenols, occasionally stained some cells of the spongy mesophyll (Figure 1j).

### 2.2. Biological and Phytochemical Surveys

**Figure 2:**
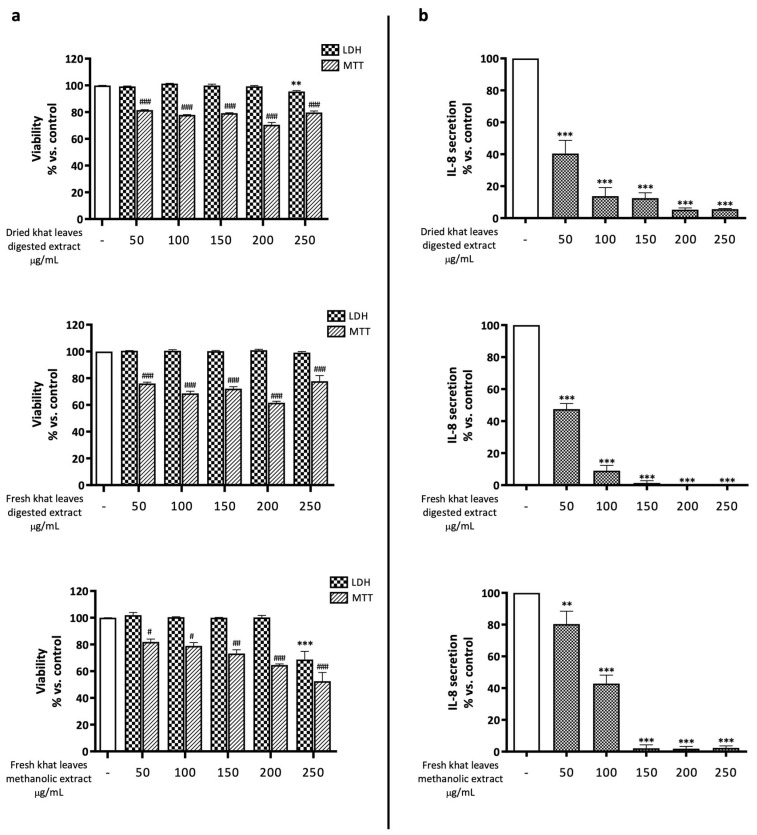
(**a**) Assessment of the effects of khat leaf extract on GES-1 viability through MTT and LDH assays. GES-1 cells were treated for 24 h in presence of increasing concentrations of extracts. Data are reported as percentages with respect to the control, which was arbitrarily assigned the value of 100%. ** *p* < 0.01; *** *p* < 0.001 versus LDH control; # *p* < 0.05, ## *p* < 0.01, ### *p* < 0.001 versus MTT control. (**b**) Assessment of the effects of khat leaf extract on IL-8 release in GES-1 cells treated for 24 h in presence of increasing concentrations of extracts. Data are reported as percentages with respect to the control, which was arbitrarily assigned the value of 100%. ** *p* < 0.01, *** *p* < 0.001 versus control.

GES-1 cells were exposed for 24 h to increasing concentrations of in vitro-digested dried and fresh khat leaf extracts (Figure 2). None of the extracts caused a significant increase in cell mortality up to a concentration of 250 µg/mL, as demonstrated by the measurement of LDH released in the culture medium. Nevertheless, a statistically significant reduction in cell metabolic activity, potentially due to reduced cell proliferation, was observed in the MTT assay at all concentrations tested. Similar results were obtained with the hydromethanolic extract, which did not alter cell mortality up to a concentration of 50 µg/mL, but, in line with previous results, determined a reduction in MTT values (Figure 2a).

The investigation of the effect on the release of IL-8 showed that both the digested and hydrometanolic extracts significantly reduced the chemokine basal secretion in a concentration-dependent manner, abolishing it at the highest concentration (Figure 2b). This data could be explained, at least in part, by the reduction in cell proliferation inferred from the MTT results. However, the strong impairment of IL-8 release observed at low concentrations suggested that khat extracts could prevent IL-8 release through anti-inflammatory mechanisms.

**Figure 3:**
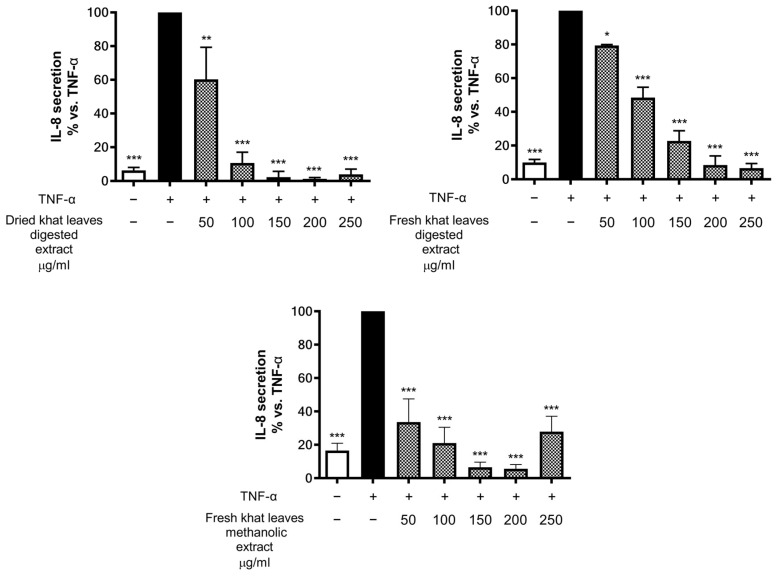
Assessment of the effects of khat leaf extract on IL-8 release in GES-1 cells. Cells were stimulated with 10 ng/mL TNF-α and treated for 6 h in presence of increasing concentrations of extracts. Data are reported as percentages with respect to the stimulated control, which was arbitrarily assigned the value of 100%. * *p* < 0.05, ** *p* < 0.01, *** *p* < 0.001 versus TNF-α.

Moving on from this evidence, the effect of the extracts on IL-8 release was further evaluated also in cells challenged with the concomitant TNF-α pro-inflammatory stimulation (Figure 3). Even in this case, all the extracts inhibited TNF-α-induced IL-8 secretion in a statistically significant and concentration-dependent manner (with minor effects on MTT assay, Figure S3), denoting the putative anti-inflammatory activity of the extract, which was expressed on a coexisting inflammatory substrate mimicked by TNF-α induction.

The quantification of the psychoactive alkaloids cathinone and cathine in the extracts yielded the following results (Table 1 and Figure S2).

**Figure 4:**
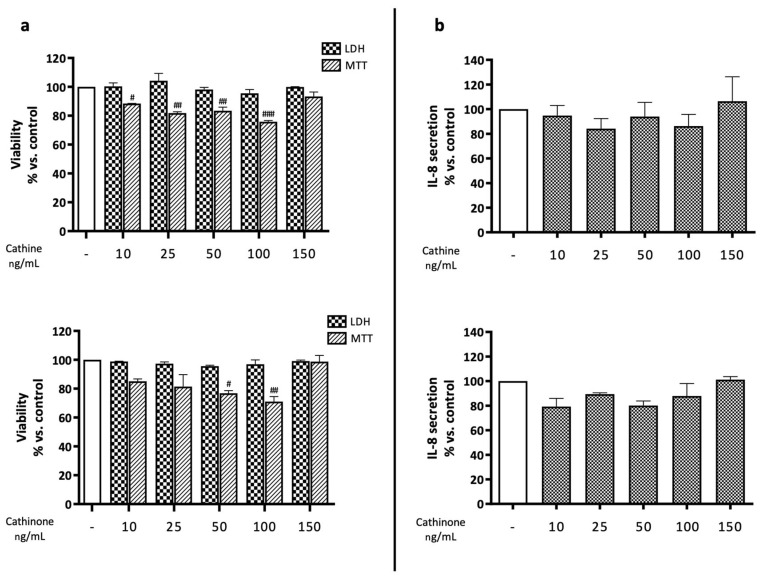
(**a**) Assessment of cathine’s and cathinone’s effects on GES-1 viability through MTT and LDH assays. GES-1 cells were treated for 24 h in presence of increasing concentrations of alkaloids. Data are reported as percentages with respect to the control, which was arbitrarily assigned the value of 100%. # *p* < 0.05, ## *p* < 0.01, ### *p* < 0.001 versus MTT control. (**b**) Assessment of cathine’s and cathinone’s effects on IL-8 release in GES-1 cells treated for 24 h in presence of increasing concentrations of extracts. Data are reported as percentages with respect to the control, which was arbitrarily assigned the value of 100%.

**Figure 5:**
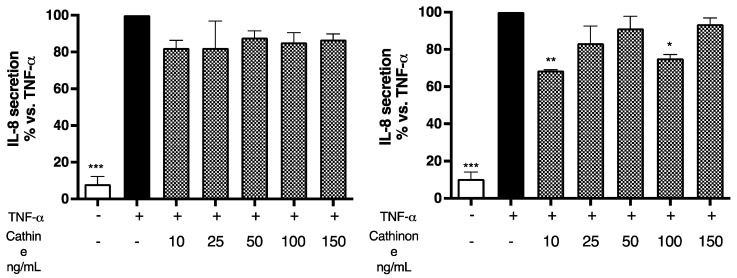
Assessment of cathine’s and cathinone’s effects on IL-8 release in GES-1 cells. Cells (basal level, white bars) were stimulated with 10 ng/mL TNF-α (black bars) and treated for 6 h in presence of increasing concentrations of cathine or cathinone (patterned bars). Data are reported as percentages with respect to the stimulated control, which was arbitrarily assigned the value of 100%. * *p* < 0.05, ** *p* < 0.01, *** *p* < 0.001 versus TNF-α.

In the extract obtained from dried vegetable material, cathinone was present in negligible amounts, confirming the literature data that report the degradation of cathinone after drying. Following the quantification, cells were treated with equivalent concentrations of the isolated molecules, falling in the range of 10–150 ng/mL, to ascertain their contributions to the total effect. Similarly to the extracts, no significant effect was observed on cell mortality; however, a partial contribution to the impairment of cell viability was attributed to both molecules, with no concentration–response fashion (Figure 4a). On the contrary, the alkaloids were unable to reduce IL-8 secretion, either in the absence (Figure 4b) or in the presence (Figure 5) of TNF-α co-stimulation, thus excluding their involvement in the previously demonstrated effects.

## 3. Discussion

Khat is known for its psychoactive effects related to the presence of the alkaloids cathinone and cathine [1,3,4,5]. Although khat chewing has cultural value for several populations, its abuse and addictive effect are a matter of concern for public health [12,13]. Moreover, results from various studies correlate the use of khat with gastroesophageal inflammatory diseases and oral neoplasia [16,17]. Intriguingly, the toxic effect of khat at the gastric level has not been validated by experimental studies; on the contrary, there is an ethnopharmacological tradition of using khat against gastrointestinal ailments [2].

Concerning the morpho-anatomy of the leaves, the literature data are scant, with the most recent contribution, showing accurate drawings of the cross-sections of the fully expanded leaves, dating back to 1962 [19].

In the present work, the leaf morpho-anatomy was described for the first time by means of light microscope images, with a comparative investigation between the young leaves and the fully expanded ones. The features described by the previous authors were confirmed [19], especially the occurrence of funnel-shaped cells in the transitional region between the palisade and spongy parenchyma and the presence of abundant druses occupying almost the entire cell volume in association with the vascular system or at the lower epidermis.

Concurrently to the anatomical survey, a histochemical approach was adopted for the first time, with special focus on alkaloids. The psychoactive substances were proven to be stored in the vacuoles of mesophyll cells at the palisade–spongy transition zone. In addition, polyphenols were occasionally detected in some cells of the spongy parenchyma.

In the present in vitro study, khat extracts obtained through simulated digestion and hydroethanolic maceration proved to be not responsible for significant cytotoxic effects in GES-1 gastric epithelial cells. Instead, the extracts proved to be able to inhibit the release of IL-8, the principal chemokine involved in gastric inflammatory states, both in basal conditions and under TNF-α or *Helicobacter pylori* stimulation [20,21]. The effect might be, at least in part, due to the reduction in cell proliferation, but we supposed that anti-inflammatory mechanisms occurred, thus demanding a specific investigation. Moreover, the results demonstrate that the preliminary anti-inflammatory effects reported in this study are not attributable to the psychoactive alkaloids found in khat leaves, cathinone and cathine, thus suggesting that extracts devoid of these alkaloids could be beneficial as anti-inflammatory agents.

Other authors have suggested that khat extracts might contain significant amounts of polyphenols responsible for inhibitory effects on nitric oxide production in macrophages [17]. However, the phytochemistry of non-alkaloid compounds from khat was poorly reported, thus encouraging further studies aimed at valorising potential alkaloid-free extracts from khat.

In conclusion, even though possible in vivo toxic effects as a consequence of prolonged use might not be excluded, the results of this study contribute to questioning of the hypothesis that khat chewing should be considered the principal cause of gastric inflammatory disorders anecdotally reported in consumers. Thus, we suggest the contribution of other confounding factors, such as the concomitant intake of alcohol and tobacco, and call for more in-depth studies to better define the biological activities of khat and achieve a balanced and empirically oriented evaluation of the plant.

## 4. Materials and Methods

### 4.1. Plant Material

*Catha edulis* (Vahl) Endl. fresh bundles were obtained from judicial seizure. The collection of the plant samples was performed concurrently for the biological, phytochemical, and morphological investigations.

### 4.2. Micromorphological Survey

This survey involved both young apical leaves and fully expanded leaves collected at different nodes along the stem to evaluate the variability of the morpho-anatomical features and to highlight the main compound classes of the secondary metabolites, with special focus on the alkaloids. The observations were carried out by means of light microscopy (LM) through histochemical procedures.

Fresh material was frozen and cryostat-cut in semi-thin sections (20–25 μm thick). The samples were also fixed in formalin–acetic acid–alcohol (FAA) solution for 7 days, embedded in historesin (Technovit 7100, Kulzer, Hanau, Germany), and ultramicrotome-cut in sections 2 μm thick. The following histochemical dyes were employed: Sudan III/IV for total lipids [22], periodic acid Schiff stain for total polysaccharides [23], calcofluor for β-glucans and cellulose [23], ferric trichloride for polyphenols [24], and Wagner’s and Dragendorff’s reagents for alkaloids [23]. Control procedures were contemporarily carried out for all the employed histochemical assays.

Observations were made with a Leitz DM-RB Fluo-optic microscope (Spectrographic Ltd., Guiseley, UK).

### 4.3. In Vitro Simulated Digestion–Extraction

The plant material was extracted fresh or after being dried for 8 h in a vacuum oven at 35 °C. A specimen of fresh khat leaves was weighed before and after being dried overnight in a laboratory oven set at 160 °C to determine the loss after drying (58.56%). Khat leaves (fresh or oven dried), were extracted with an in vitro-simulated digestion protocol that included a prolonged salivary phase (2 h), mimicking the chewing process, followed by a gastric phase, adapted from the RIVM report 320102004/2005 “Suck model” [25,26]. The duration of the salivary phase and the type of in vitro-simulated digestion were selected on the basis of literature evidence describing khat chewing behaviour [27]. In brief, an amount of material equivalent to 600 mg of dry leaves was grossly ground for 5 min in a Potter homogeniser with 10 mL of salivary buffer at 37 °C to mimic chewing. Ground leaves were transferred to a 50 mL tube, and the homogeniser was rinsed with 11 mL of salivary buffer that had been transferred to the tube. Leaves were incubated in a salivary buffer for 2 h at 37 °C on an orbital shaker to mimic the period during which the khat bolus is kept in the mouth. At the end of the salivary phase, the suspension was centrifuged at 2750× *g* for 5 min, and 18 mL of the supernatant was transferred to a new tube to mimic the ingestion of saliva produced during chewing without the ingestion of leaves. After the addition of 12 mL of gastric juice, the sample was incubated for 1 h at 37 °C on an orbital shaker. Then, the pH was adjusted to 2.5 to mimic the physiological variation in pH during digestion and incubation, and this was continued for 1 h. At the end of the gastric phase, the tube was centrifuged at 2750× *g* for 5 min. The supernatant, containing the substances that ideally come into contact with the gastric mucosa, was collected and freeze-dried (Modulyo 4K, Edwards Vacuum, Cinisello Balsamo, Italy). The same procedure was conducted without the addition of vegetal material to obtain a control blank. The lyophilised extracts were redissolved in water at a concentration of 50 mg/mL, aliquoted, and stored at −20 °C until subsequent experiments.

### 4.4. Hydromethanolic Extraction

To obtain the polar fraction of the phytocomplex, a hydromethanolic extract (MeOH 70% *v*/*v*) of fresh leaves was prepared with a drug/solvent ratio of 1:10, referring to dry leaves, compensating for the amount of water present in fresh material. Leaves were cryomacinated with liquid nitrogen, moistened with the solvent, and immediately transferred to an extraction flask. The extraction was conducted by stirring under an inert atmosphere for 6 h at 4 °C. The suspension was filtered through filter paper on a Büchner funnel to separate the liquid from the solid plant material. The filtrate was stored at -20 °C under an inert atmosphere, while the residue was extracted again overnight under the aforementioned conditions. The following day, the suspension was filtered, and filtrates were brought together. The solvent was evaporated to dryness using a rotary evaporator (Laborota 4000 efficient, Heidolph Instruments GmbH & Co., Schwabach, Germany), and the aqueous residue was freeze-dried (Modulyo 4K, Edwards Vacuum, Cinisello Balsamo, Italy). The lyophilised extract was redissolved in a mixture of 75:25 DMSO:H_2_O at a concentration of 50 mg/mL, aliquoted, and stored at −20 °C until subsequent experiments.

### 4.5. Detection of the Psychoactive Alkaloids: Cathine and Cathinone

The presence of the psychoactive alkaloids cathinone and cathine in the extracts was determined using an LC-MS/MS analytical technique. The identification of these compounds was confirmed through comparison with certified standard solutions.

#### 4.5.1. Reagents and Standards

Methanol (99.8% purity) was purchased from J.T. Baker B.V. (Deventer, The Netherlands); solutions of cathinone HCl (1 mg/mL) and cathine HCl (0.1 mg/mL) in methanol were obtained from Merck (Rome, Italy); and trifluoroacetic anhydride was acquired from SUPELCO (Messina, Italy). Diphenylamine, serving as the internal standard to adjust for instrument and extraction variabilities across samples, was also utilized (Merck, Rome, Italy).

#### 4.5.2. Sample Preparation

The lyophilised plant extracts were resuspended in methanol and subjected to overnight extraction by mixing (1000 RPM). Samples, after being centrifuged, (10 µL) were diluted with acidic water (0.2% formic acid, 80 µL) and added to internal standard (10 µL 2.5 µg/mL diphenylamine).

The analysis was performed on an HPLC Dionex 3000 UltiMate system (Thermo Fisher Scientific, Waltham, MA, USA) coupled with a tandem mass spectrometer AB Sciex 3200 QTRAP (Sciex, Milan, Italy) operated under positive ESI mode. The set parameters included a CUR of 30, GS1 and GS2 at 40, capillary voltage of 5.5 kV, and source temperature of 400 °C. Chromatographic separation was achieved on a Varian, Polaris 3 C18 ether 2.0 × 100 mm using (A) water + 0.1% formic acid and (B) methanol + 0.1% formic acid as the mobile phase. The elution program (%B) was 0–3 min 10–15%, 3–5 min 15–95%, 5–8 min 95%, and 8.0–8.2 min 95–10%, maintained until 10 min. The flow rate was 0.4 mL/min, and the column and the autosampler temperature were 30 °C and 15 °C. The comprehensive analytical data of both chromatograms and spectra were processed using Analyst software (v.1.2, Sciex, Framingham, MA, USA). Quantitative analysis was performed by means of semi-automatic area integration using MultiQuant (v.2.1, Sciex). MRM transitions were set up as follows: cathine (*m*/*z* 152 > 134, Declustering potential DP, 15 eV; collision energy CE, 13 eV), cathinone (*m*/*z* 150 > 132, DP 21 eV; CE 17 eV), and internal standard diphenylamine (*m*/*z* 170 > 93, DP 36 eV; CE 35 eV). A volume of 5 μL of extracts in water was injected into the apparatus.

### 4.6. Cell Culture

The biological activity of khat extracts was assessed in vitro on human gastric epithelial cells GES-1 (RRID:CVCL_EQ22), a transformed cell line, obtained with the permission of Dr. Dawit Kidane-Mulat (Howard University, College of Medicine, Washington D.C., USA). GES-1 cells were maintained as an adherent monostrate at 37 °C, in a 5% CO_2_ humidified atmosphere, in Roswell Park Memorial Institute (RPMI) 1640 medium (Gibco^TM^; Thermo Fisher Scientific, Monza, Italy) supplemented with 10% heat-inactivated foetal bovine serum (Euroclone, Pero, Italy), 100 U/mL penicillin, 100 µg/mL streptomycin (Pen Strep Gibco^TM^; Thermo Fisher Scientific, Monza, Italy), and 2 mM l-glutamine (Thermo Fisher Scientific, Monza, Italy). Every 4 days, at 80–90% confluence, cells were detached from 75 cm^2^ flasks (Primo^®^; Euroclone, Pero, Italy) using Trypsin-EDTA 0.25% (Gibco^TM^; Thermo Fisher Scientific, Monza, Italy), counted, and sub-cultured in a new flask at a density of 1.05 × 10^6^ cells per flask to allow for cell line expansion. For the experimental procedures, cells were seeded in 24-well flat-bottom culture plates (Falcon^®^; Corning Life Sciences, Amsterdam, The Netherlands) at a standard density of 1.55 × 10^4^ cells/cm^2^ and cultured for 72 h before the treatment.

### 4.7. Cell Treatment

To assess cell viability and cytotoxic effects, GES-1 cells were treated for 24 h with increasing concentrations of khat extracts using serum-free medium. The release of IL-8 was evaluated both in basal conditions after 24 h of treatment and in the presence of the pro-inflammatory stimulus human recombinant TNF-α (10 ng/mL; Peprotech, London, UK) after 6 h of treatment. The biological effects of the psychoactive alkaloids cathinone and cathine on the same parameters were investigated by treating cells at concentrations analogous to those detected in khat extracts.

### 4.8. Cytotoxicity Assays

The integrity of cell morphology before and after the treatments was assessed by means of light microscope inspection. The viability of GES-1 cells was investigated by MTT [3-(4,5-Dimethyl-2-thiazolyl)-2,5-diphenyl-2H-tetrazolium bromide] [28] and LDH (lactate dehydrogenase) release [29] assays.

#### 4.8.1. MTT Assay

After 24 h of incubation in the presence of increasing concentrations of the extracts, the medium was removed and replaced with 0.1 mg/mL MTT [3-(4,5-Dimethyl-2-thiazolyl)-2,5-diphenyl-2H-tetrazolium bromide] (Merck Life Science, Milan, Italy) solution in PBS, and cells were incubated for 45 min at 37 °C. At the end of incubation, the MTT solution was discarded, formazan crystals were dissolved with a 90:10 isopropanol:DMSO solution, and the absorbance was measured at 570 nm using a multilabel plate reader (EnVision 2101, PerkinElmer Italia S.p.a., Milan, Italy). This method is an undirect index of viability and proliferation since it evaluates the activity of the mitochondrial enzyme succinate dehydrogenase.

#### 4.8.2. LDH Assay

Following the treatment, the potential cytotoxic effects were determined by measuring the amount of LDH released in the medium compared to total intracellular LDH with the LDH Cytotoxicity Detection Kit (Takara Bio Europe, Saint Germain-en-Laye, France). To overcome the limitations due to the underestimation of the proportion of dead cells in conditions with growth inhibition, the modified protocol proposed by Smith et al. [29], including additional condition-specific controls, was used. After 24 h of incubation in the presence of increasing concentrations of the extracts, Triton X-100 was added to condition-specific control wells (final concentration 1%) to obtain maximal LDH release, and the plate was centrifuged at 250× *g* for 10 min. Then, 50 µL of the supernatant was taken from each well, transferred to a 96-well plate, diluted with 50 µL of water, and mixed with 100 µL of reaction mixture. After 30 min of incubation at room temperature, absorbance was read at 490 nm using a multilabel plate reader (VICTOR X3, Perkin Elmer S.p.a., Milan, Italy). The results, expressed in terms of cell viability, were calculated according to the equation modified by Smith et al. [29].

### 4.9. Measurement of IL-8 Release

The release of IL-8, the main chemokine involved in gastric inflammation, was investigated both in basal conditions after 24 h of treatment and upon TNF-α pro-inflammatory stimulation after 6 h of treatment using an enzyme-linked immunosorbent assay (ELISA) on culture media. A Human IL-8 ELISA development kit was purchased from PeproTech (PeproTech, London, UK). In brief, 96-well EIA/RIA plates (Corning Life Sciences B.V., Amsterdam, The Netherlands) were coated overnight at room temperature with a capture antibody contained in the kit. The amount of IL-8 in the samples was detected by measuring the absorbance resulting from the colorimetric reaction between an HRP-conjugate and 2,2′-azino-bis(3-ethylbenzothiazoline-6-sulfonic acid) (ABTS) substrate (Merck Life Science, Milan, Italy). Absorbance was measured at 405 nm using a multilabel plate reader (VICTOR X3; PerkinElmer, Milan, Italy). IL-8 levels were extrapolated from a standard curve of the chemokine, ranging from 0 to 1000 pg/mL.

### 4.10. Statistical Analysis

All the experiments were performed at least in triplicate. All data are expressed as mean ± SD, and the statistical significance of differences between means was calculated through unpaired one-way ANOVA followed by Dunnett’s post hoc test for multiple comparisons. Statistical analyses were conducted using GraphPad Prism 8.0.1 (GraphPad Software Inc., San Diego, CA, USA); *p* values less than 0.05 were considered statistically significant.

## Figures and Tables

**Table 1:** Cathinone and cathine quantification in khat leaf extracts.

	Cathinone (ng/µg ± SD)	Cathine (ng/µg ± SD)
Dried khat leaf digested extract	0.014 ± 0.000	0.114 ± 0.008
Fresh khat leaf digested extract	0.036 ± 0.005	0.192 ± 0.028
Fresh khat leaf hydromethanolic extract	1.327 ± 0.196	1.362 ± 0.247

## Data Availability

The original contributions presented in the study are included in the article/supplementary material, further inquiries can be directed to the corresponding author.

## Supplementary Materials

The following supporting information can be downloaded at: https://www.mdpi.com/article/10.3390/plants13111538/s1, Figure S1: Structure of the psychoactive alkaloids of *Catha edulis*; Figure S2: Chromatograms of methanolic extraction from *Catha edulis* fresh leaves (a) and its comparison with a solution of pure cathine and cathinone (b). Figure S3. Assessment of khat leaf extract effects on GES-1 viability through MTT assay. GES-1 cells were treated for 6 h in presence of increasing concentrations of extracts and TNF-α. Data are reported as percentages with respect to the stimulated control, which was arbitrarily assigned the value of 100%. * *p* < 0.05, ** *p* < 0.01 versus TNF-α.
