# Supplementary material for: Catha edulis Leaves: Morphological Characterization and Anti-Inflammatory Properties in an In Vitro Model of Gastritis
Source: Plants (Basel). 2024 Jun 1;13(11):1538. doi: 10.3390/plants13111538 (PMC11174529; doi:10.3390/plants13111538)
Supplement: Supplementary file 1 [file plants-13-01538-s001.zip › plants-3025526-supplementary.pdf]

Supplementary materials

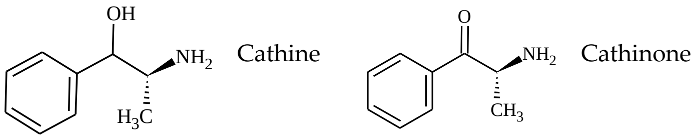

Figure S1. Structure of the psychoactive alkaloids of *Catha edulis*.

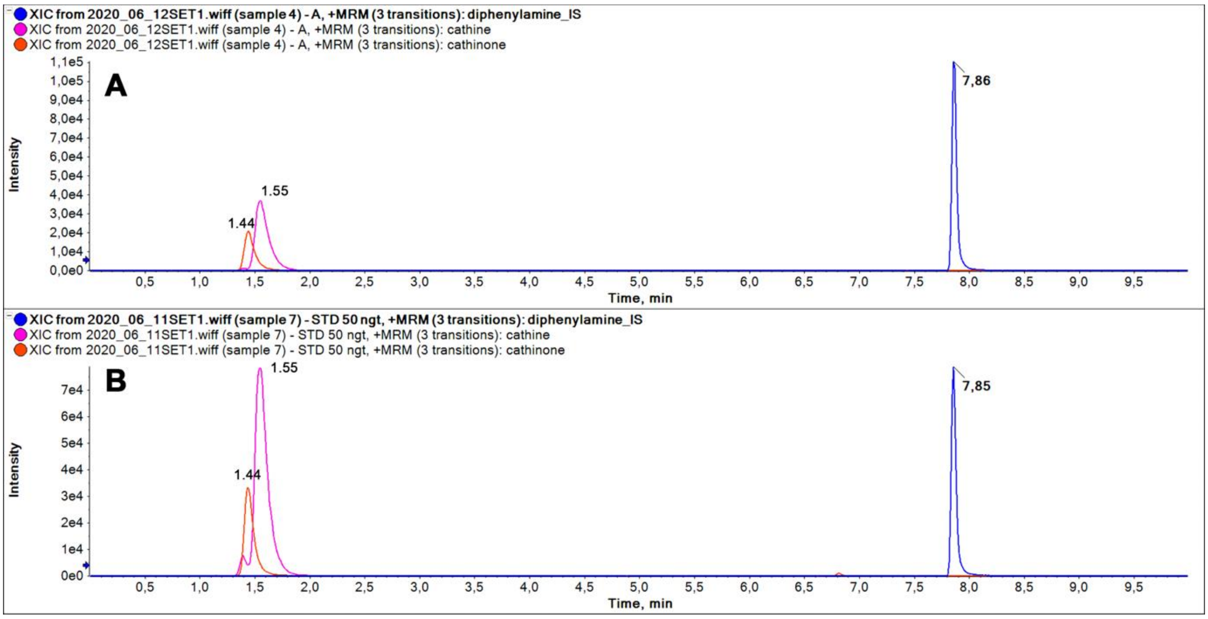

Figure S2. Chromatograms of methanolic extraction from *Catha edulis* fresh leaves (**a**) and its comparison with a solution of pure cathine and cathinone (**b**).

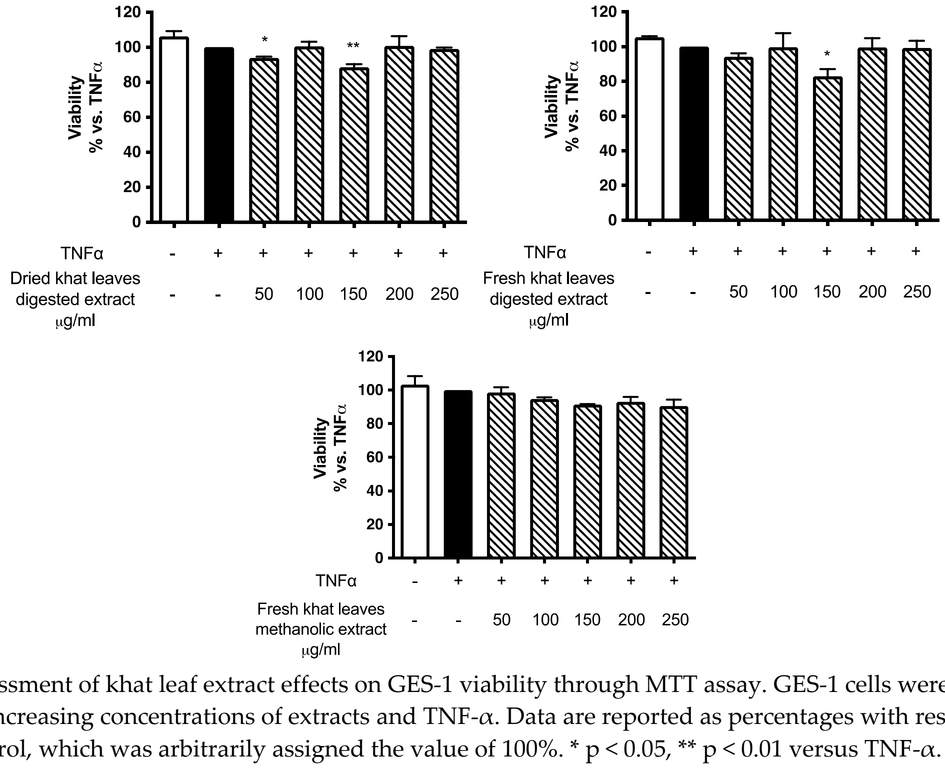

Figure S3. Assessment of khat leaf extract effects on GES-1 viability through MTT assay. GES-1 cells were treated for 6 h in presence of increasing concentrations of extracts and TNF-α. Data are reported as percentages with respect to the stimulated control, which was arbitrarily assigned the value of 100%. * $p < 0.05$, ** $p < 0.01$ versus TNF-α.
